# Molecular Docking analysis of the TNIK Receptor protein with a potential Inhibitor from the NPACT databas

**DOI:** 10.6026/97320630016387

**Published:** 2020-05-31

**Authors:** Arokiaraj Sherlin Rosita, Tajuddin Nargis Begum

**Affiliations:** 1 PG and Research Dept. of Biotechnology, Jamal Mohamed College (Autonomous), Affiliated to Bharathidasan University, Tiruchirappalli, India

**Keywords:** NPACT, TNIK, colorectal cancer, virtual screening

## Abstract

It is of interest to design and develop efficient inhibitors to the TNIK protein target in Wnt signaling pathways in the context of colorectal cancer (CRC) using molecular docking models. We show data to support that a compound named aglafoline (methyl (1R,2R,3S,3aR,8bS)-1,8b-dihydroxy-6,8-dimethoxy-3a-(4-methoxyphenyl)-3-phenyl-2,3-dihydro-1H cyclopenta [b] [1] from the NPACT (Naturally Occurring Plant-based Anti-cancer Compound-Activity-Target database) database have optimal binding features with the TNIK receptor for further consideration in this context.

## Background

Colorectal cancer (CRC) is one of the main reasons for morbidity and mortality of peoples in the world. It is the second leading cancer between females and third cancer between the males [1]. The Wnt signaling pathway plays a major role in colorectal cancer. Wnt pathway is activated abnormally in CRCs that occur sporadically and moreover, 90% of colorectal cancers and caused because of the functional loss of adenomatous polyposis coli (APC) tumor suppressor gene, which result in the constitutive activation of Wnt signaling. [2]. The genetic and epigenetic variations of colorectal cancer have been calculated widely in earlier period. The most prominent finding is, colorectal cancers hold mutations in gene that are participated into the canonical Wnt/ β-catenin signaling pathway [3]. β-catenin encoding genes like (CTNNB1), frizzled 10 (FZD10), T-cell factors 3 and 4 (TCF3/4) (TCF7L1/2), axis inhibitor 2 (AXIN2), and APC membrane recruitment protein1 (AMER1, WTX or FAM123B) are also altered repeatedly in colorectal cancer [4]. TNIK mediates proliferative Wnt signals in crypts of the small intestine and colorectal cancer cells by nuclear translocation and subsequent phosphorylation of the transcription factor TCF4 [5]. TNIK is a vital regulatory factor of Wnt signaling, and colorectal tumor cells are extremely based on the expression and catalytic activity of TNIK for proliferation [6]. TNIK has also been reported as a novel therapeutic target in several types of cancers studies, the expression of TNIK has been proved to be involved in the survival of human cancer cells which includes colorectal, gastric, liver, and hematological cancer [7]. Therefore, it is of interest to design and develop efficient inhibitors to the TNIK protein target in Wnt signaling pathways in the context of colorectal cancer (CRC) using molecular docking models.

## Materials and Methods

### Protein Preparation:

**Figure 1:**
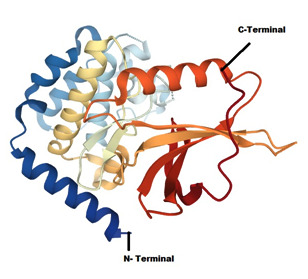
a) Molecular docking interaction of the compound (C_28_H_28_O_8_) with TNIK receptor b) Docked pose the compound (C_28_H_28_O_8_) in its binding pocket of TNIK Receptor (PDB code: 2X7F). Yellow line represents Hydrogen bond interactions.

Structure of the protein TNIK (Figure 1) was retrieved from PDB (PDB ID: 2X7F). Retrieved protein was prepared by the addition of hydrogen atoms and the removal of any heterogeneous molecules including water by using the prepare_receptor4.py script from MGLTools.

### Ligand Preparation:

NPACT (http://crdd.osdd.net/raghava/npact/) is a curated database of plant originated natural compounds that show antitumor activity. It have 1574 access and every record gives details on their structure properties (physical, elemental and topological) cancer type, cell lines, inhibitory values (IC50, ED50, EC50, GI50), molecular targets, commercial suppliers and drug likeness of compounds [8]. Among the total entries we retrieved only 543 compounds were reported for colorectal cancer. SMILES format of those compounds were taken from NPACT and convert as PDB format using Online Smiles Translator. All the compounds were loaded using input molecule option and were energy minimized with the MM2 process and changed to pdb. extension file which is readable at the ADT interface.

### Molecular descriptors calculation:

Smiles notation of ligands was used to estimate the molecular descriptors of chosen compounds using Molinspiration (www.molinspiration.com). They molecular descriptors like log P, molecular weight, polar surface area, number of atoms, number of rotatable bond, number of O or N, number of OH or NH, ion channel modulator, drug-likeness and number of violations to Lipinski's rule were calculated in the present study [9].

### ADMET prediction:

The druggability and pharmacokinetic assessment of the compound was done using pkCSM (http://biosig.unimelb.edu.au/pkcsm/) [10] and Medchem designer 3.0 software [11].

### Molecular Docking:

Molecular docking of the filtered ligands with the target site of the protein was performed with the Autodock Tools 1.5.6. The grid was fixed at the target site of the protein. AutoGrid was helped for the preparation of the grid map using a grid box. The grid size was set to 66 X 66 X 66 xyz points with grid spacing of 0.385 Å and grid center was designated at dimensions (x, y, and z): 1.085, 0.864 and 2.564. The ligands were docked into the active site of the protein where the reference ligand was bound [12]. Finally the confirmations were clustered and the poses with the lowest minimisation energy were chosen. The 3D interaction was analysed with PyMol and the 2D interaction with PoseView tool.

## Results and discussion:

**Figure 2:**
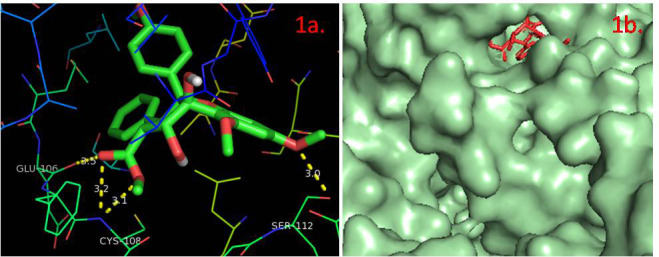
A three-dimensional cartoon representation for chain A of Crystal structure of the kinase domain of human Traf2- and Nck- interacting Kinase with Wee1Chk1 inhibitor (2X7F) [14]. The C-terminus is colored as red while N-terminus is colored as blue.

The therapeutic capacity of herbs and medicinal plants draw consideration to learn natural products as a therapeutically important source of drug molecules, they are evolutionarily optimized as drug-like compounds and remain the greatest resources of drugs and drug leads. Out of 534 compounds we have chosen 50 natural compounds based on the Lipinski's rule of five. Lipinski's rule 5 was essential to pharmacological industries to increase the activity and selection of ligand. A structure-based druggability assessment was carried-out using different stand-alone (MedChem Designer 3.0) and online tools (pkCSM). From the total availability of compounds only 50 compounds show drug likeliness, which is obtained from Medchem Designer 3.0, were shown in Table 1. Molinspiration were studied to calculate the molecular properties and drug likeness score of the selected compounds. Based on the scoring parameters we selected 2 compounds (Table 2). A molecule having bioactivity score more than 0.00 is most likely to possess considerable biological activities, while values -0.50 to 0.00 are expected to be moderately active and if score is less than -0.50 it is presumed to be inactive [13]. The selected two compounds have good acceptable range of Kinase inhibitor, GPCR, Enzymelink, Nuclear receptor ligand, ion channel modulator and protease inhibitor. Prediction of ADME properties on early stage used to avoid expensive reformulation on later. Results of pkCSM web server, confirmed that these two compounds have very good intestinal oral absorption and minimal in Blood Brain Barrier by obtaining its ADME properties (Table 3). With help of Auto dock program molecular docking was carried out. Results of this docking study showed that the compounds (C19H24O7 and C28H28O8) efficiently binds to the binding pocket of TNIK Receptor (PDB code: 2X7F) and it also gives good interaction with least binding energy and 3 hydrogen bond interactions with GLU 106, CYS 108 and SER 112. The top potential binding affinities of the (C19H24O7 and C28H28O8) with TNIK receptor binding sites was displayed in Figure 2 and their corresponding energy values were showed in Table 4 respectively. The results of the molecular docking analysis indicate that the compound C28H28O8 were more selective towards the ATP-binding pocket of TNIK Receptor. These binding energy values indicate that the new compound has shown a fortunate selectivity towards ATP-binding pocket of TNIK Receptor, which might be a reason for good activity against TNIK receptor of colorectal cancer.

### Conclusions:

We document the optimal binding features of Aglafoline (methyl (1R, 2R, 3S, 3aR, 8bS) -1,8 b-dihydroxy-6, 8-dimethoxy -3a- (4-methoxyphenyl)-3-phenyl-2, 3-dihydro-1H cyclopenta [b] [1] benzofuran-2-carboxylate from the NPACT database with the TNIK receptor for further consideration in the context of CRC.

## Figures and Tables

**Table 1:** Druggability score prediction results (Medchem designer 3.0)

S. No	Molecular Formula	M Log P	S+ LogP	S+ LogD	Rule of 5	M.wt	M.no	T_PSA	HBDH
1	C20H18O6	1.279	3.094	3.094	0	354.362	6	70.29	1
2	C29H33N3O3	2.886	4.641	3.132	0	475.635	6	69.75	3
3	C20H14O5	1.869	2.738	2.738	0	334.331	5	65.74	0
4	C20H30O3	3.803	4.745	4.745	0	318.459	3	46.53	1
5	C16H16O3	2.595	3.475	3.472	0	256.303	3	46.69	2
6	C28H28O7	2.188	3.399	0.709	0	476.53	7	105.45	3
7	C20H14O6	2.422	2.857	2.857	0	350.33	6	63.22	0
8	C25H38O5	2.7	4.475	4.475	0	418.577	5	79.29	2
9	C29H37N3O3	2.886	4.641	3.132	0	475.635	6	69.75	3
10	C15H26O	3.721	4.758	4.758	0	222.373	1	20.23	1
11	C13H14O3	2.42	3.393	3.379	0	218.254	3	46.53	1
12	C22H22O7	1.851	3.078	3.078	0	398.415	7	72.45	0
13	C20H28O4	2.28	2.525	2.525	0	332.443	4	70.06	2
14	C16H12O7	0.015	2.578	2.149	0	316.269	7	120.36	4
15	C13H12O3	1.989	2.841	2.803	0	216.238	3	50.44	1
16	C17H24O2	3.431	3.881	3.881	0	260.379	2	40.46	2
17	C15H10O6	0.525	2.201	2.079	0	286.243	6	111.13	4
18	C14H10O4	1.774	3.735	3.7	0	242.233	4	59.67	1
19	C20H20O5	3.02	3.573	3.573	0	340.378	5	46.15	0
20	C22H28O5	2.65	4.603	4.603	0	372.464	5	46.15	0
21	C18H22O9	-0.718	1.047	1.047	0	382.37	9	117.73	0
22	C19H24O7	0.272	0.908	0.908	0	364.398	7	102.43	1
23	C21H34O4	3.755	4.896	4.895	0	350.502	4	66.76	2
24	C27H36O7	1.898	3.308	3.308	0	472.582	7	110.13	2
25	C29H46O5	3.773	4.242	2.342	0	474.686	5	97.99	4
26	C28H4005	3.481	4.563	4.563	0	456.627	5	76.13	1
27	C27H3246	2.251	3.664	3.664	0	452.551	6	74.97	0
28	C15H22O5	0.708	-0.096	-0.096	0	282.339	5	86.99	3
29	C17H26O4	2.834	3.008	3.007	0	294.394	4	66.76	2
30	C14H16O4	1.875	2.491	2.444	0	248.281	4	55.76	1
31	C22H24O8	1.103	2.711	2.71	0	476.431	8	92.68	1
32	C14H16O3	2.172	2.633	2.633	0	232.281	3	35.53	0
33	C20H26O6	2.34	1.88	1.877	0	362.426	6	99.38	4
34	C23H40O3	3.647	6.578	6.578	0	364.572	3	57.53	2
35	C18H20O5	2.572	3.406	3.403	0	316.356	5	68.15	2
36	C17H34O3	2.89	3.737	3.737	0	286.458	3	60.69	3
37	C17H32O3	2.89	3.123	3.123	0	284.442	3	60.69	3
38	C19H40O3	3.504	5.259	5.259	0	31.6528	3	60.69	3
39	C17H24O3	3.556	4.229	4.228`	0	276.378	3	46.53	1
40	C28H28O8	1.707	3.255	3.255	0	492.529	8	103.638	2
41	C58H94O26	3.481	4.563	4.563	0	456.627	5	76.13	1
42	C27H32O6	2.251	3.664	3.664	0	452.551	6	74.97	0
43	C19H28O7	0.855	0.783	0.783	0	368.43	7	102.29	2
44	C6H6O3	0.086	-0.014	-0.056	0	126.113	3	50.44	1
45	C22H24O9	-0.601	2.324	2.324	0	432.43	9	94.82	0
46	C23H32O6	1.686	0.812	0.812	0	404.507	6	104.06	3
47	C21H28O7	1.129	1.504	0.504	0	392.452	7	99.13	1
48	C17H26O4	2.834	3.008	3.007	0	294.394	4	66.76	2
49	C21H2206	2.013	3.498	3.281	0	370.405	6	96.22	3
50	C18H2805	1.747	2.572	2.572	0	324.42	5	72.83	1
Notes: S+logP. LogP calculated using Simulations Plus' highly accurate internal model. S+logD. LogD at user-specified pH (default 7.4), based on S+logP. MlogP. Moriguchi estimation of logP. HBDH. A number of Hydrogen bond donor protons. M_NO. A total number of Nitrogen and Oxygen atoms. T_PSA. The topological polar surface area in square angstroms. Rule Of Five. Lipinski's Rule of Five: a score indicating the number of potential problems a structure might have with passive oral absorption. Rule Of Five_Code. Lipinski's Rule of Five codes: LP=logP; Hb=number of Hydrogen bond donor protons; Mw=molecular weight; NO=number of Nitrogen- and Oxygen-based Hydrogen bond acceptors. The presence of a code means that the corresponding Lipinski rule was violated.

**Table 2:** Bioactivity score for the selected compounds from molinspiration

S.No	Compound name	Gpcr ligand	Ion channel modulator	Kinase inhibitor	Nuclear receptor Ligand	Protease Inhibitor	Enzyme inhibitor
1	C19H24O7	0.1	0.23	0.18	0.12	0.27	0.12
2	C28H28O8	0.1	-0.07	0.09	0.47	0.01	0.03

**Table 3:** ADME/TOX Profile Prediction results

S.No	Compound name	Property name				
		Absorption Intestinal Oral absorbtion (human)	Absorption Skin	Distribution BBB	Metabolism Cyp2d6	Toxicity AMES
1	C19H24O7	95.426	-2.746	-0.81	NO	NO
2	C28H28O8	98.336	-2.435	-0.195	NO	NO
Notes: BBB-Blood Brain Barrier, CYP- cytochrome P, AMES- Ames test for toxicity

**Table 4:** Molecular docking analysis of the compound(C28H28O8)with binding pocket of TNIK Receptor (PDB code: 2X7F).

S. No	Compounds	Binding energy kcal/mol	H-bond interaction	H-bond distance (Å)	Ligand efficiency (Full Fitness) (Kcal/mol)	Inhibitory Constant (Ki)
1	C28H28O8	-15.01044	GLU 106 CYS 108 SER 112	2.5 3.2 3	-0.38	239.81
2	C19H24O7	-4.234	GLU 106 CYS 108 SER 112	2 1.8 2.8	-0.38	128.26
